# The transcriptional cofactor MIER1-beta negatively regulates histone acetyltransferase activity of the CREB-binding protein

**DOI:** 10.1186/1756-0500-1-68

**Published:** 2008-08-22

**Authors:** Tina M Blackmore, Corinne F Mercer, Gary D Paterno, Laura L Gillespie

**Affiliations:** 1 Terry Fox Cancer Research Laboratories, Division of BioMedical Sciences, Faculty of Medicine, Memorial University of Newfoundland, St John's, NL, A1B 3V6, Canada

## Abstract

**Background:**

*Mier1 *encodes a novel transcriptional regulator and was originally isolated as a fibroblast growth factor early response gene. Two major protein isoforms have been identified, MIER1α and β, which differ in their C-terminal sequence. Previously, we demonstrated that both isoforms recruit histone deacetylase 1 (HDAC1) to repress transcription. To further explore the role of MIER1 in chromatin remodeling, we investigated the functional interaction of MIER1 with the histone acetyltransferase (HAT), Creb-binding protein (CBP).

**Findings:**

Using GST pull-down assays, we demonstrate that MIER1 interacts with CBP and that this interaction involves the N-terminal half (amino acids 1–283) of MIER1, which includes the acidic activation and ELM2 domains and the C-terminal half (amino acids 1094–2441) of CBP, which includes the bromo-, HAT, C/H3 and glutamine-rich domains. Functional analysis, using HEK293 cells, shows that the CBP bound to MIER1 *in vivo *has no detectable HAT activity. Histone 4 peptide binding assays demonstrate that this inhibition of HAT activity is not the result of interference with histone binding.

**Conclusion:**

Our data indicate that an additional mechanism by which MIER1 could repress transcription involves the inhibition of histone acetyltransferase activity.

## Background

MIER1 is a newly described transcriptional regulator that functions in anterioposterior patterning in the *Xenopus *embryo [1] and as an inhibitor of anchorage-independent growth of breast carcinoma cells [2]. Two major protein isoforms, MIER1α and β, have been identified [3] and structurally, these two isoforms share a number of domains with other transcriptional regulators, including ELM2 [4], SANT [5] and acid activation domains. At the molecular level, MIER1 can both activate and repress transcription. The former involves the N-terminal acidic activation domain [6] while repression occurs by at least two distinct mechanisms: displacement of transcription factors, like Sp1, from their cognate binding sites [7] and recruitment of the chromatin remodeling enzyme, HDAC1 through its ELM2 domain [8]. Recently, studies have shown that the SANT domain also plays a crucial role in chromatin remodeling; in particular, this domain is required for efficient histone acetylation [9]. In this report, we extended our investigation of MIER1 in chromatin remodeling by examining its ability to interact with CBP and regulate its HAT activity.

## Methods

The GST fusion and myc-tagged hmi-er1β (GenBank: NM_001077701) sequences were constructed using pGEX-4T-1 and pCS3+MT plasmids, respectively and their production has been described elsewhere [7,8]. The full-length mouse CBP (GenBank: NM_001025432) in pRc/RSV was a kind gift from Dr. Roland Kwok (University of Michigan). CBP _1–1096 _and CBP_1094–2441 _were constructed by PCR amplification of the full-length sequence using 5'-ggggatccatggccgagaacttgctggacg-3' (forward) with 5'-cgggatccctacataagtgcctggcgtagctcctcg-3' (reverse) and 5'-ggggatccgcacttatgccaactctagaag-3' (forward) with 5'-ccggatccctacaaaccctccacaaactttt-3' (reverse), respectively. The PCR products were digested with BamH1 and inserted into the BamH1 site of the pCMV-Tag2B vector (Stratagene). Anti-myc hybridoma supernatant was prepared from 9E10 cells (ATCC) [10] grown in hybridoma serum-free media (Invitrogen, Inc.) supplemented with 1% OptiMab monoclonal antibody production enhancer (Invitrogen, Inc.). GST pull-down assays were performed as in [7], using 0.35 μg of GST or equimolar amounts of GST fusion proteins and 100,000 cpm of ^35^S-labeled *in vitro *translation products. Transient transfections were performed as in [8]. HAT assays were performed as in [11]; briefly, cell lysates were subjected to immunoprecipitation with the indicated antibody and the washed beads incubated with 100 nCi [^14^C]acetyl-CoA (51 mCi/mmol, Amersham), 30 μM H4 biotinylated peptide (Upstate Biotechnology Inc.) and 300 nM trichostatin A (Sigma) in HAT buffer [11] for 45 min at 30°C. The supernatants were collected and incubated with streptavidin-agarose (Pierce) at 4°C for 20 min; the ^14^C incorporated into the bound H4 peptide was determined by liquid scintillation counting.

## Results and discussion

### The N-terminal half of MIER1 interacts with the C-terminal half of CBP

**Figure 1:**
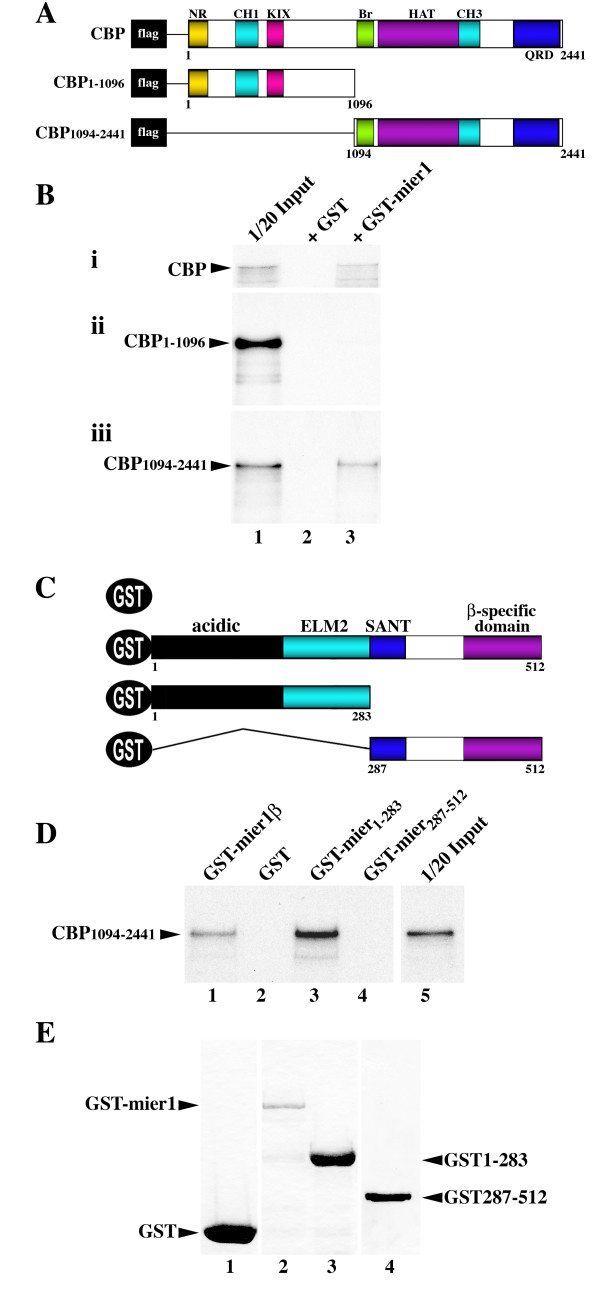
**MIER1 interacts with CBP**. (A) Schematic illustrating the CBP protein sequence and its domains: NR = nuclear receptor interaction domain, C/H1 and C/H3 = cysteine/histidine rich regions, KIX = kinase-induced interacting domain, Br = bromodomain, HAT = histone acetyltransferase domain, QRD = glutamine-rich domain. (B) GST pull-down assays using CBP deletion mutants. *In vitro *translated, ^35^S-labelled, full-length CBP (panel i), CBP_1–1096 _(panel ii) or CBP_1094–2441 _(panel iii) were incubated with 0.35 μg GST (lane 2) or an equimolar amount of GST-MIER1β fusion protein (lane 3). One twentieth of the labelled protein input is shown in lane 1. (C) Schematic illustrating the MIER1β sequence and its domains: acidic activation, ELM2 and SANT domains as well as the β-specific C-terminus. (D) GST-pull-down assays using MIER1β deletion mutants. *In vitro *translated, ^35^S-labelled CBP_1094–2441 _was incubated with 0.35 μg of GST alone (lane 2) or equimolar amounts of GST fusions of full-length MIER1β (lane 1), the N-terminal half (lane 3) or C-terminal half (lane 4) of MIER1β. One twentieth of the labelled protein input is shown in lane 5. (E) Coomassie blue-stained gel showing the GST fusion proteins used in panel D.

We investigated a possible interaction between MIER1β and CBP, using pull-down assays. ^35^S-labelled flag-tagged CBP constructs (Figure 1A), synthesized *in vitro*, were incubated with a full-length GST-MIER1β fusion protein. CBP was detected in the pull-down with GST-MIER1β, but not with GST alone (Figure 1B). Furthermore, only the C-terminal half of CBP, consisting of the bromo-, HAT, C/H3 and glutamine-rich domains, interacted with MIER1β (Figure 1B). To determine which domain(s) of MIER1β were required for binding, two deletion mutants were constructed: one consisting of the N-terminal half, which includes the acidic activation and ELM2 domains, and a second consisting of the C-terminal half, which includes the SANT domain and beta-specific C-terminus (Figure 1C). As can be seen in Figure 1D, only the N-terminal half (amino acids 1–283) of MIER1 was able to bind CBP. Since this construct contains sequence that is common to both MIER1α and β, one would expect that MIER1α would also interact with CBP. Interestingly, this region does not include the SANT domain, a domain known to play an important role in the histone acetyltransferase (HAT) activity of several chromatin remodelling complexes [12].

### Binding of MIER1 results in inhibition of CBP HAT activity

**Figure 2:**
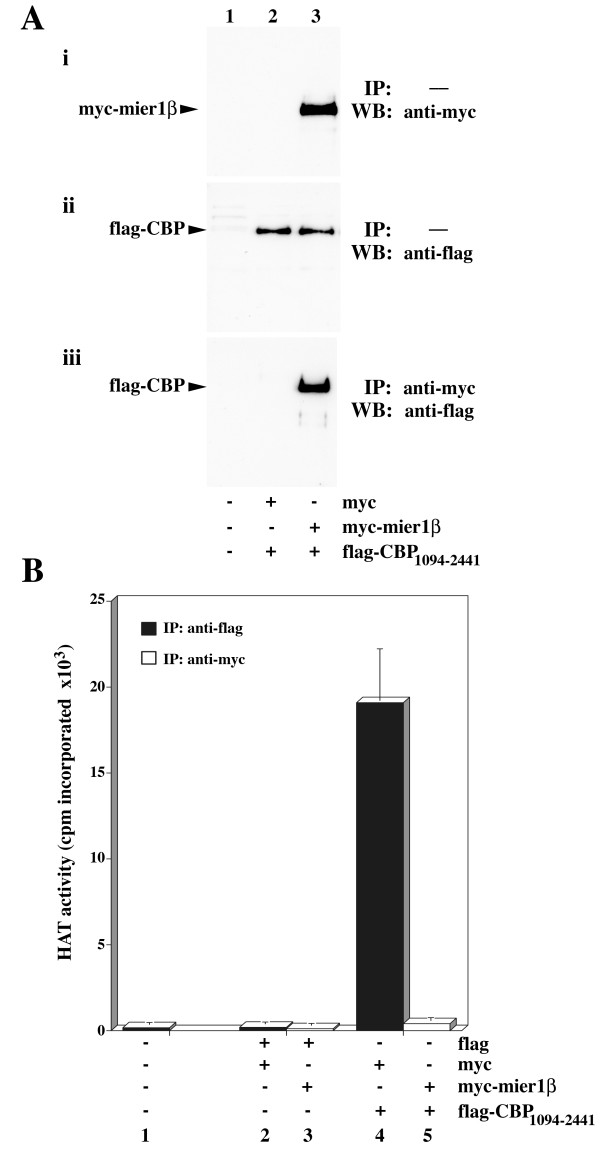
**MIER1β inhibits CBP HAT activity**. (A) Interaction between MIER1β and CBP_1094–2441 _expressed in HEK293 cells. Western blots of total extracts (panels i and ii) or anti-myc immunoprecipitates (panel iii) from nontransfected cells (lane 1) or cells co-transfected with plasmids encoding flag-tagged CBP_1094–2441 _and myc tag (lane 2) or myc-tagged MIER1β (lane 3). (B) HAT activity recovered from immunoprecipitates of nontransfected HEK293 cells (bar 1) or cells transfected with empty vectors (bar 2), myc-tagged *mier1β *(bar 3), flag-tagged *cbp*_1094–2441 _(bar 4) or flag-tagged *cbp*_1094–2441 _and myc-tagged *mier1β *(bar 5). In each sample, the total amount of DNA transfected was kept constant by including the appropriate amount of empty vector. HAT assays were performed as described in the METHODS and ^14^C-acetyl incorporation into H4 peptide was determined for each sample. Shown are the mean and standard deviation of four independent experiments.

To explore the functional consequence of MIER1-CBP interactions, we performed HAT assays on extracts from HEK293 cells co-transfected with flag-tagged CBP_1094–2441 _(flag-CBP) and myc-tagged full-length MIER1β (myc-mier1). Parallel samples were subjected to immunoprecipitation (IP) with the relevant antibody and the pellets assayed for interaction with MIER1β by Western blot or for HAT activity using ^14^C-labelled acetyl-CoA and a biotinylated histone 4 (H4) peptide. Acetylated H4 was recovered using streptavidin-agarose and the level of incorporation measured by liquid scintillation counting. Western blot analysis was used to confirm the expression of MIER1β (Fig. 2A, panel i) and CBP (Figure 2A, panel ii) in transfected cells.

As expected, no HAT activity was detectable in immunoprecipitates from cells transfected with empty vector or *mier1β *(Figure 2B, lanes 2–3), however high levels of HAT activity were measured in those from cells expressing CBP alone (Fig. 2B, lane 4). When CBP was co-immunoprecipitated with MIER1β on the other hand, no detectable HAT activity was recovered in the pellet (Fig. 2B, lane 5). The presence of CBP in the co-IP was verified in a parallel sample subjected to Western blot analysis with anti-flag (Fig. 2A, panel iii, lane 3). These data show that when associated with MIER1β, CBP has no detectable HAT activity.

### MIER1 does not interfere with histone binding to CBP

**Figure 3:**
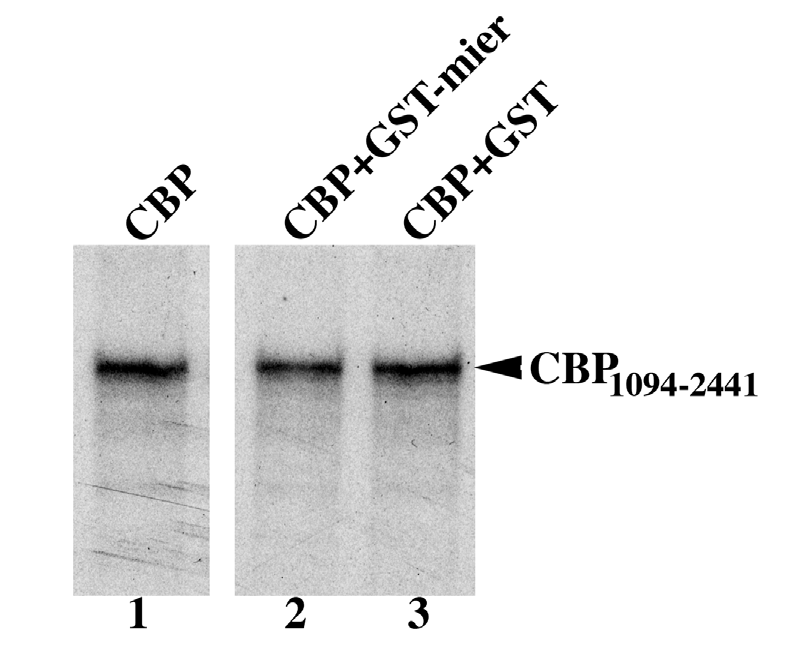
**MIER1β does not interfere with H4 peptide binding by CBP**. *In vitro *translated, ^35^S-labelled CBP_1094–2441 _was incubated with 0.1 ug biotinylated H4 peptide (Upstate Biotechnology Inc.) in the absence (lane 1) or presence of a 400-fold molar excess of GST (lane 2) or GST-MIER1β fusion protein (lane 3).

The inhibitory effect of MIER1β on CBP HAT activity could result from interference with histone binding or from a direct effect on the HAT catalytic domain. To test whether interaction with MIER1β interferes with CBP's ability to bind to histone, we measured the ability of CBP to interact with H4 peptide in the presence or absence of MIER1. *In vitro *translated ^35^S-labelled CBP_1094–2441 _was incubated with biotinylated H4 peptide in the presence of a 400-fold molar excess of GST alone or GST-MIER1_1–283 _fusion protein; the complex was precipitated using streptavidin-agarose and analyzed by autoradiography. As can be seen in Figure 3, the level of CBP associated with H4 peptide in the presence of GST-MIER1 (lane 2) was the same as that in the presence of GST alone (lane 3), demonstrating that the interaction of CBP with H4 peptide was not affected by MIER1.

Together, our data show that MIER1 physically interacts with CBP and inhibits its HAT activity; this inhibition is not the result of interference with histone binding but is possibly due to a direct effect on the HAT catalytic domain.

## Abbreviations

CBP: Creb-binding protein; GST: glutathione S-transferase; HAT: histone acetyltransferase; H4: histone 4.

## Competing interests

The authors declare that they have no competing interests.

## Authors' contributions

TMB performed the GST pull-downs and the HAT assays and participated in the interpretation of the data. CFM performed the histone binding assays and participated in the interpretation of the data. GDP participated in the design of the experiments and interpretation of the data. LLG participated in the design of the experiments and interpretation of the data, prepared the Figures and wrote the manuscript. All authors read and approved the final manuscript.
